# The application, character, and effectiveness of person-centred care with service-users, and the community within the discipline of podiatry: a scoping review

**DOI:** 10.1186/s13047-022-00566-z

**Published:** 2022-08-19

**Authors:** Sally Abey, Krithika Anil, Philip Hendy, Sara Demain

**Affiliations:** 1 Head of School, School of Health Professions, University of Plymouth, Drake Circus, Plymouth, PL4 8AA Devon UK; 2 Research Fellow, School of Health Professions, University of Plymouth, Drake Circus, Plymouth, PL4 8AA Devon UK; 3 Programme Lead: Podiatry, School of Health Professions, University of Plymouth, Drake Circus, Plymouth, PL4 8AA Devon UK; 4 Professor of Physiotherapy and Rehabilitation, Head of School of Health Sciences, School of Health Sciences, University of Southampton, Southampton, SO16 1BJ UK

**Keywords:** Scoping review, Podiatry, Patient-centred care, Patient centred-approach, Person-centred care, Person-centred approach

## Abstract

**Background:**

The concept of person-centred care is embedded within healthcare policy, focusing on long-term conditions and multimorbidity. The evidence that person-centred care is being operationalised effectively across all areas of healthcare is limited. The aim of this scoping review was to explore the application, features, and effectiveness of person-centred care with service-users, carers, and the community within podiatry.

**Methods:**

The scoping review was based upon Arksey and Malley’s five stage framework. The following databases were searched between January 2010 and March 2021: AMED, CINAHL, Embase, Cochrane library, SocINDEX, British Education Index, Business Source Complete, MEDLINE (EBSCO), and the EThOS 'Global electronic thesis and dissertation' repository, Prospero, and reference lists of included papers. Primary research articles were included if they reported on a person-centred care focused intervention with podiatry. Research terms were developed, appropriate databases identified, and an initial search resulted in 622 papers which, following removal of duplicates and critical appraisal, resulted in 18 eligible papers. Data extracted involved the types of person-centred care utilised, intervention details, motivations for engaging in person-centred care interventions, and intervention barriers and challenges.

**Results:**

Eighteen articles were included in the review. The main type of person-centred care utilised was patient/carer activities around self-management. None of the studies considered the role of the podiatrist as a person-centred care agent. The data on interventions generated the following themes ‘service facilitated person-centred care’ where a change has been made to service delivery, ‘direct clinician delivery’ where the intervention is delivered by the clinician with the patient present and ‘patient instigated participation’ where patient motivation is required to engage with an activity beyond the consultation. Outcome measures associated with quality of care and effectiveness were absent.

**Conclusion:**

There is a lack of congruency between the concept of person-centred care and how it is operationalised. A whole system approach that considers commissioning, organisational leadership, the role of the practitioners and patients has not been considered. There is immense scope for the podiatrist to play an important part in the personalised-care agenda, but currently research that can evidence the effectiveness of person-centred care in podiatry is absent.

**Review registration:**

Open Science Framework (osf.io/egjsd).

## Introduction

There is growing international agreement that implementation of person-centred care should be utilised to support patients with multimorbidity to improve quality of life and promote self-management strategies [1]. Podiatrists play an important role in monitoring, educating and treating people with long term conditions, including peripheral vascular disease (PVD), arthropathies, dementia, mental health issues, and musculoskeletal (MSK) pathology, contributing to service-user mobility [2], reducing the risk of amputation in older people with comorbidities [3, 4], and reducing foot pain [5].

The podiatrist is, therefore, well placed to engage the service-user in self-management strategies [6] and shared decision making [7]. This might include signposting to information relating to their condition and/or other healthcare services, whilst promoting positive activities related to health and wellbeing designed to encourage behaviour change. However, how the profession of podiatry currently operationalises the concept of ‘patient-centred care’ is unclear; hence, this scoping review to consider the concepts and characteristics of person-centred care outlined below [8]. The review aim is to understand the settings within which podiatrists work, their engagement with person-centred care, its effectiveness and resource implications alongside barriers and facilitators to identify gaps in knowledge.

## Review aim

The scoping review sought to examine person-centred care by podiatrists globally and identify areas where research has yet to be conducted.

### Objectives of the systematic scoping review were to:

1. identify the settings, types of person-centred care utilised, and management of those person-centred care interventions delivered by podiatrists
2. identify the effectiveness, efficiency, resourcing, and cost implications of the interventions used within a podiatry context
3. determine why service-users, carers, and communities engage with person-centred care initiatives, and the challenges and barriers that exist for all parties

## Method

### Protocol and registration

The protocol was developed using the preferred reporting items for systematic reviews and meta-analysis (PRISMA) checklist, agreed with the research team [9] and registered with the Open Science Framework retrospectively on 07.02.2021 (Registration number: osf.io/egjsd). The scoping review uses Arksey and Malley’s five stage framework to provide an overview of the literature leading to summary, synthesis and reporting [10].

### Inclusion criteria

#### Participants

The review considered articles inclusive of all ages receiving podiatry care and any duration and/or severity of a podiatry-related condition. Podiatry care was defined as care given by a qualified professional describing themselves as a podiatrist and where the standards of proficiency were considered equal to, or above, those set by the Health and Care Professions Council (HCPC) [11].

#### Intervention

Articles examining or reporting a person-centred care focused intervention relating to podiatry care were included. An article was identified as person-centred care focused if it included at least one of the following concepts as outlined in the Comprehensive Model of Personalised Care [12]:

- Seeking to enable choice (including legal right to choice)
- Supporting self-management
- Shared decision making
- Social prescribing and community-based support
- Personalised health budgets and integrated personal budgets
- Personalised care and support planning

#### Types of literature

Study designs included: randomised controlled trials (RCTs), non-RCTs, quasi-experimental, pre-, and post- studies, case studies, observational studies, systematic reviews, and qualitative studies. Opinion pieces, commentaries, book reviews, conference proceedings and non-systematic literature reviews were excluded. All articles reviewed were in English.

### Information sources

A librarian specialising in health was consulted to ensure a comprehensive review of research databases and grey literature. The searches were conducted electronically within the following databases: AMED, CINAHL, EMBASE, Cochrane database of systematic reviews, SocINDEX, British Education Index, Business Source Complete, and MEDLINE (EBSCO). United Kingdom (UK) doctoral theses were accessed via EThOS and the ‘Global electronic thesis and dissertation’ repository. A secondary search within Prospero was conducted to identify systematic reviews. Finally, the reference lists of the review papers were searched to identify further publications. The search terms used can be viewed in Appendix 1.

### Data items and data analysis mapping matrix

A data analysis mapping matrix was developed outlining the objectives of the scoping review and a data extraction table with the data types for collection. The data mapping matrix confirmed that each objective had at least one data extraction type mapped to it and indicated the types of data that might be found within the studies. A decision was made to record ‘types of person-centred care’ based upon the definitions stated by National Health Service (NHS) England within the Comprehensive Model of Personalised Care [12]. These definitions are not used beyond England but provide categories for the types of person-centred care using an easily interpreted description.

### Study selection and data extraction table

**Fig. 1:**
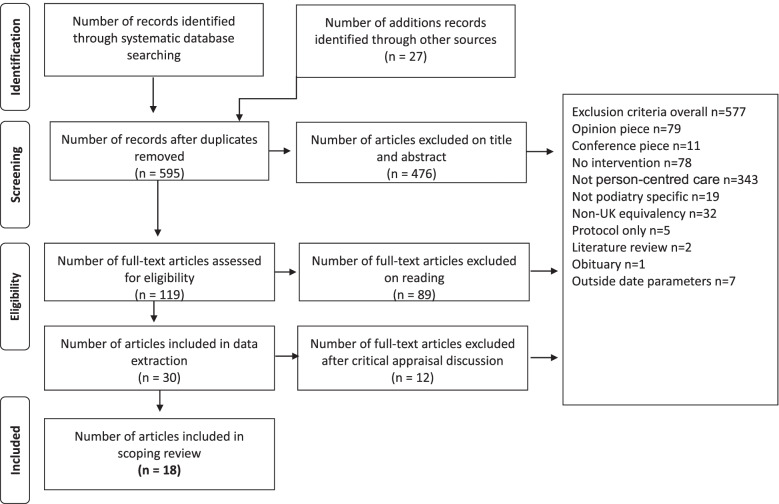
PRISMA flowchart

Citations, abstracts, and full-text papers were independently reviewed by three investigators (SA/PH/KA) against the inclusion criteria. Any disagreements arising between reviewers were resolved through discussion. The study selection process is displayed in the PRISMA flow diagram (Fig. 1).

The data extraction table was piloted to agree definitions and data extracted as follows: geographical location, setting, study aim, research methodology and demographic data of the recipient group. Intervention characteristics and outcomes including intervention type, delivery format, management of the intervention, evidence of effectiveness, efficiency, resourcing, cost implications, reasons for engagement, challenges and barriers encountered, and conclusions were also recorded.

No standardised definition exists for self-care and self-management and is used interchangeably in the literature to refer to separate concepts [13]. We define self-care as activities that promote general health (physical and mental) based on the individual’s choice, whilst self-management are activities guided by the support of others such as family or a health professional.

### Quality judgement

The Hawker et al. [14] critical appraisal tool was used to systematically assess research articles with differing study designs to assign a total score based on the quality of each research article. This was divided into category scores of low (9 to 17), fair (18 to 26), and high (27 to 36).

## Results

### Description of studies and their characteristics

Following a search of the databases, 622 citations were identified with a further 27 yielded from additional sources. Duplicates resulted in 54 citations being removed leaving 595 from which a further 119 were excluded following review of the title and abstract. Searches were concluded by 1.3.21. Upon full text reading a further 89 papers were removed and a further 12 removed upon critical review by authors, KA, PH and SA leaving 18 papers included in the scoping review (Fig. 1). The primary reasons for excluding studies at the full-text reviewing stage were: conference abstracts only available, no intervention, not related to person-centred care or did not meet the standards of proficiency set by the HCPC [11]. Table 1 displays the characteristics of the studies included in this review. However, information, such as age or gender are missing. The missing data was either not reported by the study or not applicable.

**Table 1:** Study traits/mapping

		**Intervention**					**Intervention Comparator**				
**Study**	**Condition**	**Overview**	**N**	**Age**	**Gender**	**Avg. Condition Duration**	**Overview**	**N**	**Age**	**Gender**	**Avg. Condition Duration**
Baba et al. 2015 [15]	Diabetes	Written and illustrated foot education	78	69.5	Males 52.6 Females 47.4	12.2 years	Group foot education session with audio-visual tools and led by a qualified educator	76	66.3	Male 67.1 Female 32.9	9.4 years
Creagh 2015 [16]	Diabetes	Service changes (e.g., diabetes foot care hotline, simplification of foot care pathway, etc.)	140	Not reported	Not reported	Not reported	Previous service	Not reported	Not reported	Not reported	Not reported
Noble 2019 [17]	General	Development of a self-referral system	Not reported	Not reported	Not reported	Not reported	NA	NA	NA	NA	NA
Ploderer 2018 [18]	Diabetic foot ulcers	Self-care mobile phone app	11	43–74	Males 10 Females 1	> 3 months	NA	NA	NA	NA	NA
Distiller 2010 [19]	Diabetes	Service changes (diabetics care now full responsibility of the doctor and not the service)	2726	29.6	Males 49.3% Females 50.7%	16.6 years	NA	NA	NA	NA	NA
Aard 2011 [20]	Diabetic foot ulcers	Education and caretaker monitoring	Not reported	Not reported	Not reported	Not reported	NA	NA	NA	NA	NA
Spink 2011 [21]	Falls and disabling foot pain	Multifaceted podiatry care (e.g. provision of footwear, education, exercise programme, etc.)	153	74.2	Males 47 Females 106	6.1 years	Routine podiatry care	152	73.6	Males 47 Females 105	7.7
Farndon 2018 [4]	Peripheral arterial disease	Podiatry-led integrated pathway	21	Not reported	Males 15 Females 6	Not reported	NA	NA	NA	NA	NA
Farndon 2016 [22]	General (Podiatrists also included)	Self-management online toolkit for foot wear	Patients 13 Podiatrists 6	Not reported	Not reported	Not reported	NA	NA	NA	NA	NA
Keukenkamp 2018 [23]	Diabetic foot ulcers	Education and motivational interviewing	5	57 (median)	Males 5	29 years (median)	Education	5	62 (median)	Males 4 Female 1	17 (median)
Kileen 2019 [24]	Diabetic foot ulcers	Remote temperature monitoring	4	68	Males 4	Not reported	NA	NA	NA	NA	NA
Williams 2014 [25]	Visual impairment and diabetes	Diabetes self-management education and non-visual foot exam	52	Not reported	Not reported	Not reported	Usual foot examination by person with sight	Not reported	Not reported	Not reported	Not reported
Grimmer-Sommes 2010 [26]	Diabetes (GPs also included)	Service changes (e.g. Integration of GPs in private practice and free access to AHPs)	Patients 59 GPs Approx. 74	Not reported	Not reported	Not reported	NA	NA	NA	NA	NA
Chuter 2019 [27]	NA^a^	Service examination (e.g. podiatry services, educational resources, education / training programmes)	Not reported	Not reported	Not reported	Not reported	NA	NA	NA	NA	NA
Hu 2019 [28]	General	Holistic chronic disease self-management and rehabilitation program	294	52.40	Males 114 Females 180	Not reported	Usual care	521	47.06	Males 181 Females 340	NA
van Netten 2019 [29]	Diabetic foot ulcers	Foot self-care Education and management	Not reported	Not reported	Not reported	Not reported	NA	NA	NA	NA	NA
Navarro-Flores 2015 [30]	Diabetes	Education and self-care	Not reported	Not reported	Not reported	Not reported	NA	NA	NA	NA	NA
Heng et al. 2020 [31]	Diabetic foot ulcers	Collaborative education	33	55.2	Females 14	14.7	Traditional education	19	60.1	Females 2	16

*N* = Denotes total sample size for each condition; some conditions included more than one type of participant group (e.g. patients and GPs), we have specified the participants groups and respective sample size where needed

^a^Study examined podiatry services, not individuals with a health condition

### Geographical distribution of studies

**Fig. 2:**
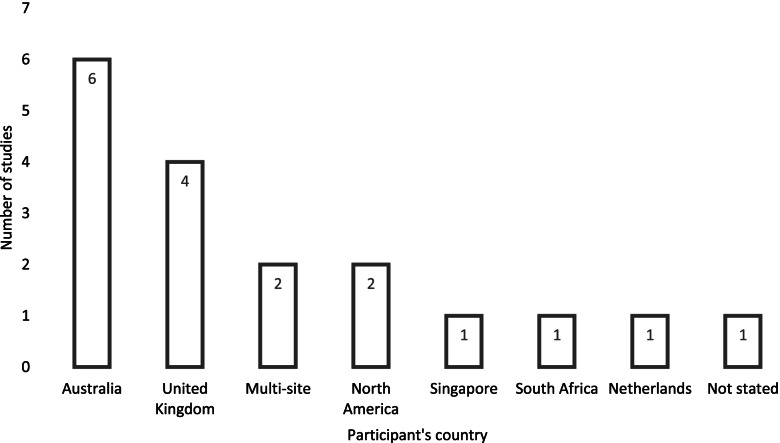
Geographical distribution of studies

Geographical distribution represents countries where data were collected for each study (Fig. 2). Of the 18 studies, 11% were multi-sites. Australia [15, 18, 21, 26–28] contributed 33% of the papers and the United Kingdom 22%, representing 55% of the research undertaken in the area of person-centred care.

### Setting/context

A variety of settings were represented with 41% at multiple sites [4, 16, 21, 26, 27, 29, 30]. Community settings accounted for 27% [15, 17–19, 28] with a further 6% in the home [25], and 16% at podiatry clinics or medical centres [23, 24, 31]. Eleven percent of the studies did not state the context or setting of the study [28, 29].

### Study aims

Diabetes was the main focus of 72% of the studies reviewed [15, 16, 18–20, 23–25, 27, 29, 30], with 6% focusing on patients with PVD [5], 6% on patient with chronic disease [28] and 12% on those with visual impairment [24, 25]. None of the studies specifically used the term person-centred care (or a similar term) within their aims. There was, however, reference to ‘foot self-care and self-exam’ by one study [30], but these terms were not defined. ‘Foot examination’ using touch and smell alongside usual care was clearly outlined by one study [25]. The aims relating to diabetes varied from reducing amputation rates 6% [16], prevention, or early identification, of ulceration at 22% [20, 24, 29, 30]. One study looked at the prevention of falls in an older population with disabling foot pain (6%). Comparison of effectiveness of different education methods in relation to changes in foot health behaviours and attitudes was considered by one study (6%). Two studies (11%) focused specifically on Aboriginal and Torres Strait Island people. Chuter et al. [27] undertook a systematic review to consider ‘programmes’ which had successful outcomes in terms of foot related complications due to diabetes and Hu et al. [28] considered the differences between those who undertook a programme designed to support better chronic disease management and those who did not. Four studies (22%) undertook a service or pathway development approach [4, 17, 19, 26] in relation to how the service was operationalised [17, 19, 26], the feasibility of an integrated service [4] and cost-effectiveness and service improvement benefiting patients and clinicians [17].

### Intervention focus

The scoping review considered who the person-centred care intervention was aimed at, which was categorised as ‘person/carer’ (56%) [15, 18, 20, 21, 23–25, 29–31], ‘practitioner’ zero studies, and ‘service’ one study [19]. The remaining studies combined ‘person/carer’ and ‘practitioner’ (6%) [22], ‘person/carer’ and ‘service’ (17%) [4, 17, 26] or all three categories (17%) [16, 27, 28].

### Intervention types employed

The data extracted was analysed using descriptive qualitative analysis [32, 33] and initially coded by author, SA. Emergent categories were noted and subsequently checked for coherence by the authors, KA and SA (Table 2) leading to three overarching categories.

**Table 2:** Frequency table of the interventions utilised

Category	Sub-category	Frequency
Service facilitated person-centred care	Referral pathways to access assessment/care Multidisciplinary approaches Clinician empowerment	2 2 1
Direct clinician participation	Teaching via educator Self-care reminders Standard monitoring / treatment protocols Referrals to promote health change behaviours Motivational interviewing Education digitally-based	6 1 4 3 2 1
Patient instigated participation	Self-care Education paper-based Telehealth Non-visual foot exam	3 2 3 1

### Category 1: service facilitated person-centred care

This theme describes a concept where an intervention made a structural change to service delivery for the purposes of person-centred care and has three sub-themes. The sub-theme ‘referral pathways to access assessment/care’ describes increased access to a range of services for patients with diabetes [16] and improvements for access to podiatry services via self-referral [17]. The sub-theme ‘multidisciplinary approaches’ describes changes to care delivery where a group of healthcare professionals combined their expertise for the assessment and treatment of an individual patient [16, 29]. ‘Clinician empowerment’ was represented by one study which described giving more control over prescribing choices to clinicians to improve patient outcomes [19]; however, this paper did not specify from where power was transferred.

### Category 2: direct clinician participation

The theme ‘direct clinician participation’ describes a concept where the intervention is directly delivered by the clinician to the patient and has six sub-themes. This could be a treatment intervention, a person-centred care activity or referral to another clinician based on the patient’s needs. It is an activity that is instigated and led by the clinician. The sub-theme, ‘teaching via educator’, includes clinicians giving educational information to patients during consultations, and educational sessions/groups set up outside of the consultation [15, 20, 23, 30]. ‘Self-care reminders’ describes a mobile phone app reminding patients to engage with self-care activities [18]. ‘Standard monitoring and usual treatment protocols’ captures studies where participants received their usual care alongside the study intervention [20, 21, 27, 29]. ‘Referrals to promote health change behaviours’ describes those studies which included interventions designed to promote healthy behaviour changes such as smoking cessation, weight loss and exercise regimes [4, 26, 28]. The sub-theme ‘motivational interviewing’ represents two interventions: one utilising motivational interviewing [23] and one utilising motivational interviewing plus focused counselling to influence self-care behaviours [31]. ‘Education digitally-based’ describes online education utilising a web-based online toolkit for supporting informed footwear choices [22].

### Category 3: patient instigated participation

‘Patient instigated participation’ reflects interventions where patient initiation was required outside of the influence of the clinician and has four sub-themes. The sub-theme ‘self-care’ represents those studies which required the patient to undertake self-care of their feet between consultations [15, 29, 30]. Two studies [16, 21] incorporated paper-based education resources such as leaflets, and are represented in sub-theme ‘education paper-based’ [15, 16]. The ‘telehealth’ sub-theme includes mobile phone apps, and the use of a temperature mat to detect daily changes in foot temperature [18, 24]. ‘Non-visual foot exam’ represents one study focusing on individuals with significant sight problems utilising smell and touch to identify potential foot issues [25].

### How interventions were delivered and types of person-centred care

It was not always clear how interventions were delivered (28%) [4, 20, 25, 27, 30]. Five of the studies (28%) provided ‘face to face education’ [19, 23, 26, 29, 31], with three (17%) providing ‘educational literature’ such as leaflets [15, 16, 21], and one study using audio-visual sessions (digital education) [15]. ‘Health technology’, such as mobile phone apps [18], an online toolkit [22] and remote temperature measuring [24] were utilised by 17% of studies. One study utilised a ‘prescribed therapy’ such as orthotic issue combined with a footwear voucher [21] with patients undertaking a ‘self-administered’ exercise programme at home [21]. Finally, one study used an online referral system to deliver the intervention [17].

Types of person-centred care were identified based on the definitions outlined previously: supports self-management (78%) [15, 16, 18, 20–31], personalised care and support plans (17%) [4, 19, 22], enabling choice (6%) [17], and shared decision making (6%).

### Types of method and data collection utilised

Systematic reviews (22%) [20, 27, 29, 30] and randomised controlled trials (22%) were the most utilised methods. The types of RCT implemented differed across studies, such as ‘parallel group randomised controlled trial’, ‘pilot randomised controlled trial design’ and ‘quasi-randomised trial’ [21, 23, 25, 31]. The most utilised primary data collection method were questionnaires (28%) [4, 15, 17, 28, 31] and observing health data (28%). Three studies used a combination of diaries, focus groups, questionnaires and observation of health data [4, 25, 28].

### Main findings of the included studies

The main findings centred around four areas: improvements in participants’ health status, health behaviour change, clinician practice changes, and improved service delivery. It is important to note that these findings should be approached with caution due to methodological flaws as revealed by the Hawker tool (see ‘Quality assessment of included studies’ section).

The majority of the studies reported improvements in participants’ health status in the following areas: reduced amputations [16], reduced admission rates [19] number of days hospitalised [16], better metabolic control for those with type 1 and 2 diabetes [4, 19] and delayed microvascular complications [19]. A reduction in falls was seen in older people suffering with severe foot pain [21] with increased patient satisfaction scores relating to a new diabetic foot service [16].

Health behaviour change was identified in 28% of the studies. Participants with diabetic foot ulcers reported they engaged more in their ulcer care using the mobile phone app [18], foot temperature monitoring was found to be beneficial in preventing foot ulceration [20], and non-visual foot checking was higher than conventional foot checking techniques in the visually impaired [25]. Collaborative approaches to education, include active listening and recognising the patient as the expert in their own care, increased knowledge retention and self-care behaviours utilising counselling and motivational interviewing strategies as part of the education [31]. Finally, a systematic review by Navarro Flores et al. [30] suggests that behavioural changes such as promoting better hygiene habits, moisturizing, selecting the correct type of shoes and adequate foot care, support better metabolic control in diabetes and reduction in amputation rates.

Clinician practice changes characterised 18% of the studies, with two focusing on educational changes and one on motivational interviewing. The educationally centred studies reported that effective foot care education required both written and audio-visual elements [15], and that footwear advice required inclusion of four aspects: practicalities, personal choice, purpose and pressure [22]. Motivational interviewing was used to support footwear adherence, however, this produced only a short term clinically relevant effect (not statistically significant) increase after one week, with levels returning to baseline over time [23].

Improved service delivery represented 18% of the studies. Improved accuracy in patient triage and patient satisfaction [17] was observed in one service evaluation, with increased quality of life scores for those with PVD following the introduction of a new integrated pathway pilot study [4]. Evaluation of an integrated service, with access to allied healthcare professionals at no extra cost, was made available to those with type 2 diabetes, which was felt by patients to increase their knowledge and to support adherence to self-manage their condition [26]. Finally, a systematic review to identify the provision of programmes seeking to reduce diabetic foot related complications in Aboriginal and Torres Islander Australians found that no such programme existed [27].

Three (17%) of the studies produced findings that had more than one element of the categories outlined above. The use of technology to support self-monitoring using thermometry requiring both a change in clinician practice and behaviour change in the patient. This was a small case series pilot study where the initial findings were positive. Three of the four cases developed ulceration which was detected using thermometry, suggesting requirement for a larger scale research project [24]. A further study considered the introduction of a self-management of chronic disease programme for Aboriginal and Torres Islander Australians (service change) demonstrating increased uptake of other healthcare services by the participants (behaviour change [28]). However, the outcome of these contacts with other healthcare services and information regarding the patients’ disease progression is not reported. Finally, a systematic review determined a strong relationship between the use of thermometry and therapeutic footwear in the prevention of recurrent foot ulceration, but that the evidence for some other widely used practices, such as foot-related exercises, single sessions of education and foot surgery to reduce ulcer recurrence was weak [29].

### Outcome measures

Pre/post data outcome measures were used by 44% of the studies using tools such as: the Nottingham assessment of functional foot care [15], amputation rates and number of days in hospital [16], pain and function sub-scales [21], quality of life surveys [4, 28], the Edinburgh intermittent claudication questionnaire [4], footwear adherence and step count [23] a post-intervention survey of an online referral system [17] and knowledge, self-care and self-efficacy behaviour questionnaires [31]. A further 33% studies recorded biomarkers such as body mass index (BMI), haemoglobin A1c (HbA1c) and muscle strength [4, 19, 21, 23, 24, 28]. Four studies (22%) used a combination of biomarkers and pre-post data outcome measures. Those studies undertaking systematic reviews (22%) produced a narrative synthesis [20, 27, 29, 30], and 16% of studies solely used qualitative data [18, 22, 26]. One study undertook a baseline ‘podiatric check’ and repeated this at three and six months, but no details of the assessment were provided [25].

### Effectiveness of intervention

Effectiveness was viewed as a measure of the extent to which a specific intervention achieved its aim for a specified population. A range of disparate interventions were reported in nine studies and are shown in Table 3.

**Table 3:** Summary of interventions and their outcome used in the included studies

Intervention	Brief Outcome
Patient education [13]	Improved foot health
Mobile app that monitored ulcers [14]	Improved ulcer care but usability and accuracy require further development
Multifaceted podiatric approach [15]	Reduced fall rates
Direct treatment from podiatrists [5]	Podiatrists can successfully provide vascular assessment and person-specific advice on lifestyle changes
Remote temperature testing for selfcare activities [26]	Supported self-care activities and improved identification of individuals requiring podiatric treatment
Non-visual foot inspection for vision impaired self-care [24]	Increased likelihood of reporting a new foot problem to a podiatrist
Motivational interviewing [25]	Short-term effectiveness
Patient education, counselling, and motivational interviewing [27]	Increased knowledge retention and self-care behaviours, reducing need for additional podiatry clinic time
Development of a toolkit [28]	Podiatrists in partnership with patients identified and addressed potential barriers to changing footwear

Interventions impacting directly on services represented 28% of the studies. Effectiveness was reported in all cases in the following areas: reduced amputation rates [16], improved quality of care [16], reduced cost to services [16], improved patient access to appropriate care [17, 26, 28], increased patient knowledge and disease awareness [26], reduced hospital admission rates [19], and improved patient satisfaction [16].

A systematic review focusing on patient education and regular monitoring treatment found these interventions to be mostly ineffective in prevention of diabetic foot ulcers, although plantar foot temperature guided avoidance therapy was reported as having potential utility due to the robust study design [20]. Arad et al. (2011) conducted a systematic review examining the effectiveness of foot health programmes to reduced diabetic foot related complications for Aboriginal and Torres Strait Island people and found inconclusive findings due to a lack of clarity of some studies reviewed [27]. A systematic review by van Netten et al. (2016) supporting the use of specific self-management and footwear intervention for the prevention of recurrent plantar foot ulcers was identified [29]. Strategies aimed at behavioural changes were found to be effective for the metabolic control of diabetes and reduction of amputations, but methodological flaws suggested that the effectiveness should be treated with caution [30].

### Efficiency and cost effectiveness of intervention

Efficiency was defined as reducing the cost of delivering a service as a direct result of the intervention. Most studies did not discuss efficiency (78%). One service improvement plan reported multiple interventions leading to a decrease in the number of days in hospital for patients and a 45% reduction per year in major and minor amputation rates resulting in a £300,000 saving per annum [16]. Another service improvement project improved triage via self-referral and reported the potential for reduced service costs and savings in relation to general practitioners (GPs) not being consulted unnecessarily [17]. Distiller et al. [19] reported reduced rates of admissions for patients with diabetes but no related cost savings. Primary care integrated service with allied professionals and GPs was reported as ‘cost efficient’ [26]. Of the 18 studies reviewed only one study provided costs relating to a footwear subsidy valued at $A100/£65 [21].

### Reason for participant engagement with studies

No explanations were provided.

### Barriers/challenges to participation with person-centred care

Fifty-six percent did not report any challenges or barriers to participation [15, 17, 19, 20, 22, 24, 26–28, 30]. Low participation engagement reported by 28% [4, 16, 23, 25, 29] and 17% ceased participation due to death, injury, illness, and lack of time to engage [4, 21, 31]. Attrition was described as ‘external issues to the study’ by 6% [23], difficulties using technology by 6% [18], and a fear of developing wounds whilst undertaking prescribed exercise 6% [23]. Of these studies, 11% reported more than one of these experiences occurring [4, 23].

### Study funding

Overall, there were 30 grants awarded across the studies. Of the studies reviewed 61% [4, 15, 18, 20, 21, 26–28] received funding, 6% [24] stated that they received no funding and 33% [16, 17, 19, 23, 25, 30] did not make a statement about funding. None of the studies stated the value of the funding received. There were three main funding streams: biopharmaceutical or health related companies, Universities, and Government enterprises. Company funding was the most utilised, with 17 separate grants [15, 20, 29, 31]. One study accounts for 12 of the grants within this category [29]. Five grants came from University funding [18, 21, 22, 27]; one study gained two grants from different faculties [28]. A further five studies received Government funding [4, 15, 21, 26, 29]. Of those studies, one received three Government funded grants [4] and another received two grants [29].

### Future research

Six studies (33%) did not discuss areas for future research or gaps in research [4, 15, 17, 18, 20, 24, 27], and 6% study stated nil research gaps [16]. Further investigation, development of the research question or understanding of the mechanism of effect was cited by 33% [4, 23, 26, 28–30] and 17% suggested further testing with a different population [20], setting [21] or larger cohort [24]. Development of technology (6%) [18] and testing the effectiveness of a toolkit developed during the study (6%) [22] was also cited.

### Authors’ conclusions from the included studies

Some authors’ reiterated their findings (61%) [4, 15, 17–20, 24, 27–30]. A further 33% added to their findings by discussing the potential for podiatrists to reduce falls [21], implementation of motivational interviewing for patients with diabetes [23] and teaching non-visual foot examination for the visually impaired [25]. Three studies commented upon the podiatrist’s role in; increasing patients making the right footwear choices [4], potential for employers to provide training to increase effectiveness of patient education to increase self-management [31]; and understanding complex relationships with other healthcare professionals as part of an integrated model of care [26]. Finally, one study commented on the need for more than service structures to be in place to reduce amputation rates but did not elucidate further [16].

### Quality assessment of included studies

Using the Hawker disparate data tool [14] the majority of the studies were deemed fair quality (53%), 29% high quality and 18% studies low quality. Studies considered “fair” was based upon a lack of consideration for ethics and potential bias in their study design, sampling reporting and where statements of generalisability were unclear. Coherent abstracts and titles, clear reporting of findings and implications led to ‘high’ categorisation. Studies categorised as “low” lacked information/clarity in their abstract, methods, sampling, analysis, generalisability, ethics and bias consideration.

## Discussion

The aim of this scoping review was to illuminate to what extent person-centred approaches have been implemented, the types of interventions utilised and researched by podiatrists globally to make recommendations for future research.

None of the 18 studies reviewed used the term ‘person-centred care’ or an analogous phrase within their aims. Most of the studies focused upon patients with diabetes. However, PVD, lower limb MSK conditions and long-term conditions other than diabetes represent a significant proportion of the caseload for a podiatrist [34, 35], but were poorly represented. The study aims relating to diabetes concentrated on reduction of amputation rates 6% [16] prevention, or early identification of, ulceration at 22% [20, 24, 29, 30]. Only one study focused on MSK aspects of the role, considering the prevention of falls in an older population with disabling foot pain [21], one study focusing on PVD [4] and another on long-term conditions [28].

Interestingly, none of the studies focused interventions on the practitioner alone, but two studies did consider the use of motivational interviewing [23, 31], focusing on the success of motivational interviewing as an intervention in relation to footwear adherence [23] and self-care behaviour changes [31]. However, to deliver person-centred approaches to care, the practitioner’s own ideology, drive for fostering a change in practice and commitment to partnership working [7, 36, 37] is critical. Research that explores skills and attitudes required by the podiatrist to practice person-centred care and the support required by senior management is currently absent from the literature.

The intervention types employed (see Table 2) proved interesting in relation to the agency of the clinician and/or patient and three overarching themes were developed. The second theme, ‘direct clinician participation’ describes the clinician’s opportunity to influence the partnership by engaging with, or referring, the patient during the consultation or continuing to influence the patient outside the consultation with self-care reminders. Although there is no guarantee that the clinician’s interventions will result in patient behaviour change, there is potential to moderate and adapt their own behaviours in response to the feedback from the patient. However, none of the studies considered the clinician’s role as an influencer within the patient/carer dynamic. There was no discussion around patient choice, skills and confidence within a person-centred care framework, or shared decision making. Only one study considered patient knowledge and how it links to patient outcomes [31]. Overall, the function of the podiatrist’s role in practicing person-centred care approaches to care remains unclear.

The third theme, ‘patient instigated participation’ captures those interventions that require patient motivation to engage with activities that may directly or indirectly impact on their health such as phone apps to measure wounds and temperature mats to detect temperature changes. The temperature mat study [24] detected temperature changes in three of the four participants resulting in ulcer detection and early treatment. A further study [25] explored the use of non-visual foot examination for the visually impaired with diabetes. These studies support further investigation to understand the utility for self-management and better patient outcomes.

The methodologies and data collection methods used both quantitative and qualitative approaches, but there is scope to conduct research in the area of podiatry using a mixed methodology with the focus on the podiatrists’ attitudes, demonstrable behaviour and skill in the area of person-centred care. Understanding the experience for the patient and researching the impact on health behaviour change and outcomes is critical to developing a clear understanding of the profession’s status and impact in this area.

Various outcome measures were utilised in eight pre/post study designs. However, outcome measures such as the patient activated measures (PAM) [38], patient reported outcome measures (PROMs) [39], and patient reported experience measures (PREMs) [40], which are associated with measuring the quality and effectiveness of person-centred approaches to care [38], were absent from these studies with limited use of quality of life (QoL) measures. These types of measures support increased communication between the patient and clinicians and are important for improving processes and clinical outcomes based upon evidence.

Motivational interviewing was present in just two of the studies [23, 31], despite its utility for supporting behaviour change [41] and other strategies that can be used by the healthcare professional such as shared decision making [12], illness integration support [41], and guided self-determination [41, 42] were all absent. Co-production [12], peer-support [12, 43], health coaches [44] were also absent from the studies. Due to the lack of clear aims or clear outcome measures, over a quarter of the studies could not be judged in terms of effectiveness.

## Clear recommendations for future research

This scoping review offers some insight into examining and implementing person-centred care in the discipline of podiatry. This review indicates that the podiatry profession requires research that focuses on a whole system approach, inclusive of commissioning, leadership and infrastructure, and podiatrist skills development as deliverers of personalised care recognising the patient’s role in the partnership. There is potential to extend the focus beyond diabetes, consider behaviour change in the patient and include outcomes that measure quality and effectiveness. Specific areas for future research should include the identification of areas of person-centred care where podiatrists can contribute most effectively, such as the development of tools that support podiatry related person-centred care which could include podiatry psychometric instruments, foot examinations with the patient’s perspective represented around facilitators and barriers, use of telehealth and other health technologies. This should be augmented by utilising mixed methodology approaches with a focus on the podiatrists’ attitudes, demonstrable behaviour, and skill in the area of person-centred care, which are areas yet to be explored. The evidence base for behaviour change, such as using motivational interviewing, understanding patient attitudes towards podiatry care should be increased. There is also a requirement for research that explores skills and attitudes required by the podiatrist and the support offered by senior management.

## Limitations

The scoping review only included papers written in English which may have excluded relevant papers published in another language. A UK-centric definition of person-centred care was used that may have skewed the results since we included studies from other countries. Additionally, studies from countries that did not meet the UK podiatry standards were excluded, possibly resulting in some relevant data being excluded from this review. The database searches were comprehensive, but the authors recognise that some articles pertaining to person-centred care could have been missed resulting in loss of relevant data. No standard framework, such as the Template for Intervention Description and Replication (TiDIER) framework [45], was used to extract data from the studies as no appropriate framework was found to address the review’s objectives. Finally, intervention effectiveness was limited to a narrative analysis since this is a scoping review.

## Conclusion

The scoping review illustrates that a research gap exists between the concept of person-centred care and its operationalisation. Research to date has tended to focus on discrete areas of activity around self-care, without regard to the whole-system within which that activity was delivered. The lack of outcome measures that are associated with quality of care, improved health outcomes and effectiveness fails to offer the profession evidence to support the development of person-centred care practice in podiatry that could be used to influence commissioners, organisations, practitioners, and patients. Podiatrists’ client base includes those with long-term conditions and multimorbidity providing an opportunity to contribute significantly to the aims of the personalised-care agenda, by supporting self-management, employing shared decision making, and by engaging with social prescribing and community based-support.

## Data Availability

The datasets used and/or analysed for this study are available from the corresponding author upon reasonable request.

## Appendix 1

**Table:** Search terms using Boolean phrases

Search terms will include ‘**AND podiat***’	
1.patient-focus* care	Synonyms for approach to practice
2.person-focus* care	
3.patient-cent?d care	
4.person-cent?d care	
5.patient-cent?d practice	
6.person-cent?d practice	
7.patient-centric	
8.Community based care	
9.Family cent?d care	
10.Relationship cent?d care	
11.Patient-led care	
12.Individuali?ed care	
13.Universal personali?ed care	
14.Self-manage* care	Education interventions
15.Self-manage* Educat*	
16.Self-care	
17.Patient educat*	
18.Service-user educat*	
19.Public educat*	
20.Community educat*	
21.Patient activation measure	Outcome measures used as baseline
22.PAM?	
23.Patient reported outcome measure*	
24.PROM?	
25.Patient cent?d outcome*	
26.PCOM?	
27.Patient reported impact measure*	
28.PRIM?	
29.Patient reported experience measure*	
30.PREM?	
31.Motivational interview*	Interventions
32.Support planning	
33.Enabling choice	
34.Personal health budget	
35.Integrated personal budget	
36.Co-produc*	
37.Peer support*	
38.health coach*	
39.group activit*	
40.asset-based approach*	
41.social prescri*	
42.shared decision making	
43.shared decision-making	
44.SDM	
45.Behavio?r chang*	
46.Making every contact count	
47.MECC	
